# Patient Beliefs and Experiences of Adhering to Medical Therapies for Cardiovascular Comorbidities, Before Noncardiac Elective Surgery in South Africa: A Mixed-Methods Study

**DOI:** 10.1213/ANE.0000000000008110

**Published:** 2026-07-03

**Authors:** Oluwanilo Awoloto, Hilary L. Bekker, Christella Alphonsus, Simphiwe Gumede, Khulile Singata, Elzanne Jansen van Rensburg, Simon J. Howell, Bruce Biccard

**Affiliations:** From the 1 School of Medicine, University of Leeds, Leeds, United Kingdom; 2 The Division of Psychological and Social Medicine, Leeds Institute of Health Sciences, University of Leeds, Leeds, United Kingdom; 3 Department of Anaesthesia and Perioperative Medicine, Groote Schuur Hospital, Cape Town, South Africa; 4 Faculty of Health Sciences, University of Cape Town, Cape Town, South Africa; 5 Leeds Institute of Medical Research, University of Leeds, Leeds, United Kingdom; 6 Nuffield Department of Clinical Neurosciences, University of Oxford, Oxford, United Kingdom.

## Abstract

**BACKGROUND:**

Cardiovascular comorbidities increase perioperative risk in patients undergoing noncardiac surgery. Optimizing medication adherence in this population is critical; yet adherence remains low in many low- and middle-income countries (LMICs), where health beliefs, systemic barriers, and healthcare communication bring significant challenges. This study aimed to explore the acceptability of validated patient-reported questionnaires and to investigate the beliefs and experiences influencing adherence to cardiovascular medication before surgery in a South African tertiary hospital.

**METHODS:**

A mixed-methods study was conducted using semistructured interviews with 21 patients taking cardiovascular medication before vascular surgery. Participants completed four validated self-report questionnaires: Brief Illness Perception Questionnaire (BIPQ), Beliefs about Medicines Questionnaire (BMQ), SHARED decision-making questionnaire, and SURE decisional conflict scale. Questionnaire responses were summarized with descriptive statistics. Thematic analysis of interview data was conducted using Braun and Clarke’s six-step framework.

**RESULTS:**

Most participants perceived their illness as chronic (15/20, 75%) and their treatment as effective (18/21, 86%), yet 15 of 21 (71%) reported high concern and 9 of 21 (43%) reported emotional distress. Belief in the need for medication was strong (21/21, 100%), but 14 of 21 (67%) worried about long-term side effects and 10 of 21 (47%) described their medication as a “mystery.” Shared decision-making appeared limited: only 7 of 21 (33%) recalled being asked for their views. Thematic analysis revealed seven major themes: understanding illness and treatment, health professional interactions, service access, social support, individual factors, routines, and habits.

**CONCLUSIONS:**

Patient-reported tools were acceptable and highlighted complex, interrelated factors associated with adherence. Integrating these tools into preoperative care may help identify at-risk patients and support person-centered perioperative planning in LMICs.

**KEY POINTS**

- **Question:** Can established patient self-report questionnaires capture patient beliefs about illness, medication, and healthcare decisions in a South African population?
- **Findings:** Using a mixed-methods design, combining questionnaires and semistructured interviews, we identified that individual, relational, and system-level factors influence how patients engage with chronic disease management in the perioperative setting.
- **Meaning:** Integrating outcomes from this study into preoperative care may help staff identify at-risk patients and support more person-centered perioperative planning in South Africa and other low- and middle-income countries (LMICs).

## BACKGROUND

Approximately 310 million major surgeries are performed annually worldwide.^1^ A key indicator of treatment effectiveness is the 30-day postoperative mortality rate.^2^ These rates vary across services and countries. The African Surgical Outcomes Study (ASOS) 2018 found that African patients are twice as likely to die following elective surgery when compared to a global cohort.^3^ Identifying what service delivery and patient factors are associated with postoperative mortality is essential to improving surgical practices and patient outcomes.

Patients with cardiac comorbidities undergoing noncardiac surgery are at increased risk of postoperative morbidity and mortality.^4^ Ensuring patients adhere to their medication regimens is key to optimizing cardiac disease management and reducing perioperative risks.^5^ Nonadherence to medication for long-term conditions in sub-Saharan African countries is as high as 62.5%.^6^ Variation in adherence has been associated with patient belief systems including traditional medicines and religious beliefs, financial constraints and sharing of medications, poor knowledge and unrealistic expectations of therapeutic interventions, and dissatisfaction and difficulties with clinical services.^7–9^ Understanding factors impacting medication adherence in the perioperative period is important to supporting patients in managing their cardiovascular comorbidities before noncardiac surgery, as therapy withdrawal has been associated with increased mortality.^10^

Established psychological models such as the common-sense model of illness provide a useful framework for considering the types of cognitions, motivations, and emotions important to people when managing their illness.^11^ Patient self-report questionnaires have been developed to explore systematically illness-related beliefs and behaviors,^12–15^ and patient involvement in healthcare decisions^16,17^ necessary to develop interventions to improve health services and patient outcomes.^18^ It is unclear if these self-report questionnaires are acceptable to patients and professionals within the African environment and could be integrated within noncardiac surgical services.

This study aimed to explore the experiences of patients with cardiovascular comorbidities toward medication adherence before noncardiac surgery at a large, government-funded, teaching hospital in South Africa. The study objectives were to first assess how acceptable established patient self-report questionnaires are for capturing beliefs about illness, medication, and healthcare decisions within a South African population. Second, it sought to explore patients’ perspectives on managing their cardiovascular disease, along with their experiences of preparing for noncardiac vascular surgery.

## METHODS

### Design

A survey design using a semistructured interview method to investigate patient beliefs and experiences about their health problems, treatments, and experience of care when presenting for noncardiac vascular surgery. The standards for reporting qualitative research (SRQR) reporting guidelines were followed.^19^ Ethical approval for this study was obtained from the Human Research Ethics Committee of the Faculty of Health Sciences, University of Cape Town (ref. no. 330/2022). Participation in this study was voluntary, and written informed consent was obtained from all subjects before participation.

### Context

This study was carried out in Groote Schuur Hospital, a tertiary teaching hospital in Cape Town, between July and August 2022. Groote Schuur is a major referral center in the Western Cape, South Africa. An initial period of observation was undertaken by O.A. to gain insight into how this type of study could be integrated within cardiovascular and surgical clinics in this large, urban hospital. The first week of the study was spent observing staff and patient activities in theaters, wards, and clinics, and talking with research, management, administrators, and clinical staff about care protocols.

During this study preparation phase, the study materials were translated from English to isiXhosa and Afrikaans (Supplemental Digital Content 1, Documents S1–S3, https://links.lww.com/AA/F793), to meet the differing needs of this culturally diverse population. Clinical teams and research nurses identified points in this vascular surgical care pathway where patients could be approached to participate in the study.

### Participants

Eligibility included adult patients presenting to the hospital for vascular surgery, who were taking cardiovascular medications and had arterial disease. Exclusion criteria included patients presenting for cardiac surgery or surgery for venous disease. Participants who were interviewed were offered an expense payment of Rs. 100.

### Study Size

We estimated a purposive sample of about 20 participants selected from vascular surgical ward rounds and clinics would provide a range of experiences to address this study’s aims.^20,21^ Three interviewers O.A., K.S., and E.J.v.R. worked with clinical teams to identify patients’ representative of across various categories of sex, age, health problem, and residential area.

### Data Collection

A survey made up of several questionnaires (listed below) was used to guide a subsequent semistructured interview exploring the participant’s (a) understanding of the questionnaire items, and (b) views and experiences about their medications and the impact of their illness on their life. The survey used the following questionnaires:

- Brief Illness Perception Questionnaire (BIPQ^22^): eight items assessing people beliefs of their illness, specifically perceived consequences, timeline (acute-chronic), amount of perceived personal control, treatment control, identity (symptoms), concern about the illness, coherence of the illness, and emotional representation.
- Beliefs about Medicine Questionnaire (BMQ^12^): 18 items assessing people’s beliefs toward medicine in general, and their medication.
- Decisional conflict screening scale (SURE^16^): four items assessing the patients’ decision, understanding of the information, recognizing risks and whether they were supported in making their choice.
- People’s experience of making a shared decision with their health professional (SHARED^17^): 10 items assessing the patient’s understanding of the information provided during the consultation, the questions they asked, and their agreement with the choice made.

### Procedure

In-patients were approached following vascular surgical consultant-led ward rounds and out-patients were approached while in the waiting areas of vascular clinics and informed about the study. Participants provided informed consent in their preferred language (isiXhosa, Afrikaans, or English), but most chose to participate in English. Those who were unable or did not want to read the questions themselves but wished to participate were given the option of having one of the interviewers read out the questions and fill in their answers for them. After completing the survey, participants were asked to indicate on the survey or verbally if they wished to participate in the follow-up interview.

Interviews were carried out at the bedside of participants or in nearby clinic rooms, conducted by O.A., K.S., or E.J.R. They were informed of confidentiality before the interview started and gave verbal consent for the interview to be recorded using a hand-held voice recorder. All interviews started with the interviewer asking if anything was unclear in the surveys; this was then explored before the semistructured interview. Recordings were translated (where applicable) and transcribed externally by a third-party.

### Analysis

Descriptive statistics were used to summarize participant responses to the questionnaires. Responses were presented as by-item frequency data to illustrate patterns of responses. Questionnaire data were managed in Microsoft Excel 2024 version 16.82.

**Table 1.** Participant Characteristics

Characteristics	N (%)
Sex	
Males	10 (48)
Females	11 (52)
Comorbidity	
Hypertension	16 (76)
Hypercholesterolemia	13 (62)
Peripheral vascular disease	10 (48)
Diabetes mellitus	14 (68)
Heart disease	4 (19)
Kidney disease	1 (5)
Number comorbidities	
1	1 (5)
2	6 (29)
3+	11 (64)
Duration of illness (y)	
>1	4 (2)
5–10	5 (24)
>10	8 (38)
Family history of cardiovascular disease	
Yes	18 (86)
Smoking	
Current smoker	11 (52)
Former smoker	6 (29)
Alcohol	
Little to none	15 (72)
3 or less days per week	3 (14)
4 or more days per week	3 (14)
Marital status	
Married	8 (38)
Divorced	5 (24)
Single	5 (24)
Widowed	5 (24)
Residence	
Township	7 (33)
City	11 (53)
Rural	3 (14)
Religion	
Christian	14 (67)
Islam	7 (33)
Employment	
Yes	5 (24)
Languages spoken	
Afrikaans	15 (71)
isi-Xhosa	2 (9)
English	20 (95)
Education level, N (%)^a^	
General	7 (33)
Further	10 (48)
Higher	3 (14)
No formal education	1 (5)
Healthcare payment, N (%)	
Government/employer	15 (71)
Self-funded	6 (29)

a South Africa’s National Qualifications framework has three broad categories of education: general (high school/vocational training, up to 16 y old); further (college diplomas/technical qualifications, 17–18 y old); higher (university degrees, 19+ y old).

**Figure.**
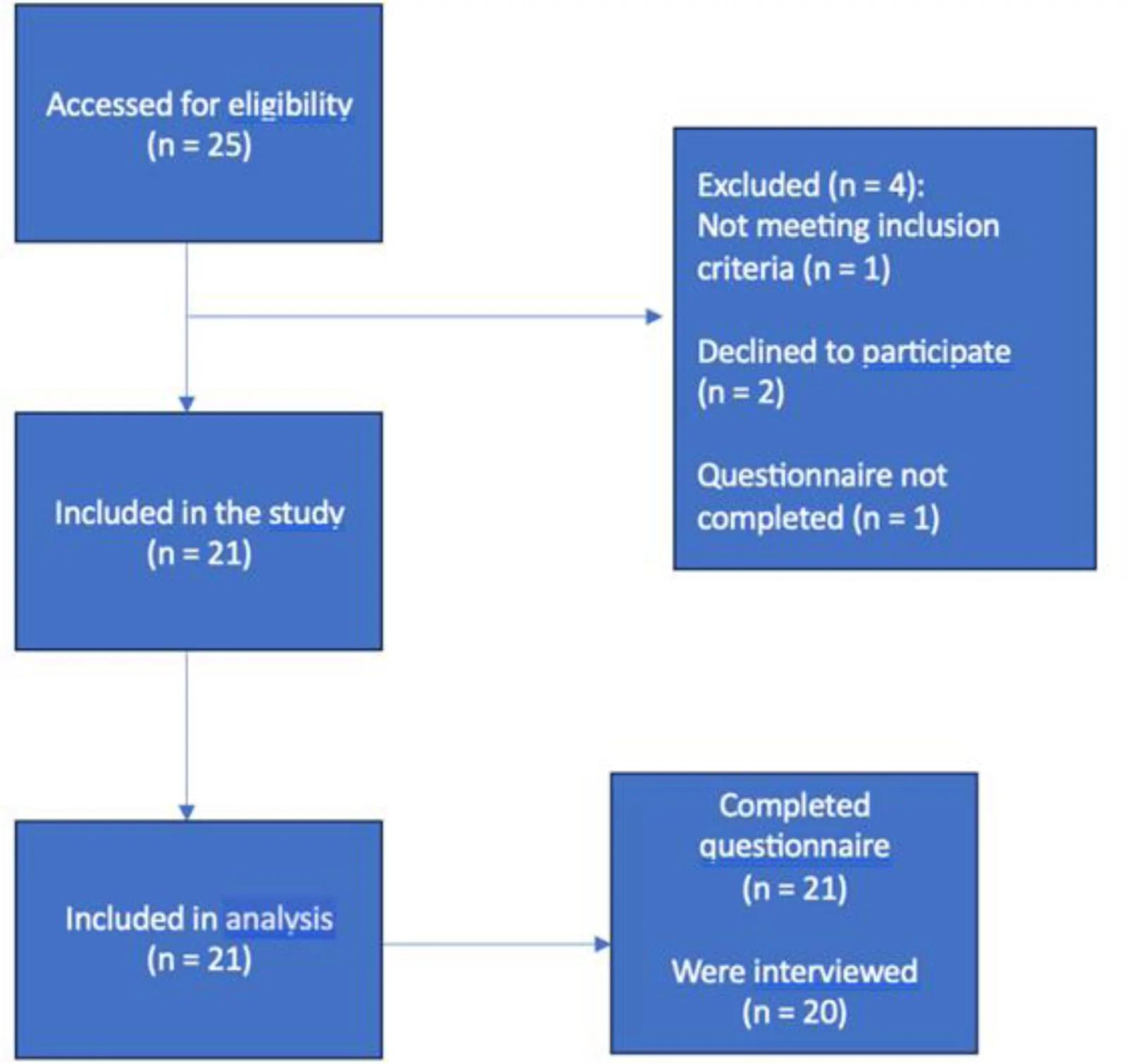
Recruitment and participant flowchart.

Interview data were transcribed into Microsoft Word and analyzed using an iterative process described by Braun and Clarke’s 2006^21^—Step 1: O.A. used three transcripts of varying lengths to familiarization herself with the text, and classify utterances to develop codes of categorizations; Step 2: coding of utterances and discussion of codes with H.L.B., S.J.H., B.B., C.A. using 15 transcripts; Step 3: generation of themes, categorization of codes within themes and discussion of categories O.A. with H.L.B., S.J.H., B.B., C.A., using previous transcripts; Step 4: reviewing of themes, and subthemes by O.A.; Step 5: naming of themes O.A. with H.L.B., S.J.H., B.B., C.A. Following this process, the remaining five transcripts were analyzed, and quotes were placed into the pre-formed themes and subthemes (O.A., H.L.B.); Step 6: the themes are presented within the Results section to illustrate the usefulness of the questionnaires to participants and synthesize participant findings about medication, adherence and involvement in their care with health professionals (all authors).

## RESULTS

Of the 21 participants, 20 took part in the interviews (Figure). Participant characteristics are displayed in Table 1. About half the participants were male (mean age of 60 [standard deviation {SD}, 9.3]). Female participants had a mean age of 61 (SD, 14.8). Approximately one-third of participants had a general level of education (high school or vocational training), half lived in the city, three-quarters were unemployed, most spoke English and Afrikaans, all followed a religion, most smoked or had smoked, and for most, healthcare was paid through government funding. All participants had a range of long-term comorbidities, predominantly hypertension, diabetes, and hypercholesterolemia.

### Questionnaires

Most participants found the questionnaires simple to understand and complete. The total participant number is 21 unless otherwise stated. Responses to the BIPQ (Table 2) showed that most participants perceived their cardiovascular disease as chronic (15/20, 75% scoring 7–10) but also reported a strong sense of personal control (14, 67%) and high confidence in treatment efficacy (18, 86%). The majority (18, 85%) felt they understood their illness well. Despite these perceptions, concern about the illness was high (15, 71%), and a substantial proportion reported significant emotional impact (9, 43%) and symptom burden (8/20, 40%). These findings suggest that while patients are generally engaged with managing their condition, many experience considerable emotional distress and concern.

**Table 2.** Brief Illness Perception Questionnaire

	N (%)		
BIPQ items	0–3 Little	4–6 Some	7–10 A lot
How much does your illness affect your life?	6 (29)	7 (33)	8 (38)
How long do you think your illness will continue?	0	5 (29)	15 (71)
How much control do you feel you have over your illness	3 (14)	4 (19)	14 (67)
How much do you think your treatment can help your illness?	0	3 (14)	18 (86)
How much do you experience symptoms from your illness?	7 (33)	5 (29)	8 (38)
How concerned are you about your illness?	4 (19)	2 (10)	15 (71)
How much do you feel you understand your illness?	1 (5)	2 (10)	18 (85)
How much does your illness affect you emotionally? (eg, does it make you angry, scared, upset, or depressed?)	8 (38)	4 (19)	9 (43)

Adapted from Broadbent et al.^22^

Responses to the BMQ (Table 3) indicated participants believed in the necessity of their prescribed treatments, with all agreeing that their current health depends on medication and 90% (19) believing their future health would as well. Most participants felt that without medication they would become very ill (19, 90%) or that life would be impossible (16, 76%). Despite this, many expressed concerns about long-term use, including worries about side effects (14, 67%) and dependence (12, 57%), and just over half believed most medicines are addictive (11, 52%). Nearly half (10, 47%) described their medication as a “mystery,” suggesting gaps in understanding; the majority (15/20, 71%) did not view it as disruptive to daily life. Participants expressed skepticism about prescribing practices, with 67% (14) agreeing that doctors might prescribe less if given more time with patients.

**Table 3.** Beliefs About Medicine Questionnaire Frequency Responses by Item

BMQ items	N (%)		
	Agree	Not sure/uncertain	Disagree
My health at present depends on my medication	21 (100)	0	0
Having to take medication worries me	6 (29)	1 (5)	14 (66)
My life would be impossible without my medication	16 (76)	1 (5)	4 (19)
Without my medication I would be very ill	19 (90)	1 (5)	1 (5)
I sometimes worry about the long-term effects of my medication	14 (67)	3 (14)	4 (19)
My medication is a mystery to me	10 (47)	1 (5)	10 (48)
My health in the future will depend on my medication	19 (90)	1 (5)	1 (5)
My medication disrupts my life	4 (19)	2 (10)	15 (71)
I sometimes worry about becoming too dependent on my medication	12 (57)	2 (10)	7 (33)
My medication protects me from becoming worse	17 (81)	3 (14)	1 (5)
Doctors use too many medicines	8 (38)	3 (14)	10 (48)
People who take medicines should stop their treatment for a while every now and again	5 (24)	3 (14)	13 (62)
Most medicines are addictive	11 (52)	1 (5)	9 (43)
Natural remedies are safer than medicines	9 (43)	4 (19)	8 (38)
Medicines do more harm than good	2 (10)	3 (14)	16 (76)
All medicines are poisons	3 (14)	3 (14)	15 (72)
Doctors place too much trust on medicines	9 (43)	4 (19)	8 (38)
If doctors had more time with patients, they would prescribe fewer medicines	14 (67)	4 (19)	3 (14)

Adapted from Horne et al.^12^

**Table 4.** SHARED: Patient Experience of Shared Decision-Making Questionnaire (SHARED-Q10) Frequencies

SHARED-Q10 items	N (%)		
	Agree	Not sure	Disagree
The doctor…			
talked about other options from the one I chose	6 (29)	4 (19)	11 (52)
talked about whether or not there was a medically best option for my health problem	10 (48)	1 (4)	10 (48)
gave their views about the options	10 (48)	2 (9)	9 (43)
asked for my views about the options	7 (33)	1 (5)	13 (62)
I talked about…			
what was important to me about this decision	10 (48)	1 (4)	10 (48)
why one option suited me better than another	9 (43)	1 (4)	11 (52)
the risks and benefits of the options for me and my health	7 (34)	1 (4)	11 (52)
I felt…			
it would be OK to choose any option we talked about	5 (23)	6 (29)	10 (48)
the decision made was the best one for me	16 (76)	5 (24)	0
the doctor and I agreed which option was the best one for me	10 (48)	3 (14)	8 (38)

Responses to the SHARED Questionnaire (Table 4) suggest that while most participants felt positively about the outcome of their treatment decisions, key elements of shared decision-making were often lacking. Although 76% (16) agreed that the decision made was the best one for them, fewer reported having discussed alternative options (6, 29%), the risks and benefits of treatment (7, 33%), or their own preferences and values (10, 43%). Only one-third (7, 33%) recalled being explicitly asked for their views. Overall, these findings highlight a gap between patient-reported satisfaction with decisions and their experience of a collaborative decision-making process.

### Thematic Analysis of Interview Responses

The named themes identified from participant responses when exploring issues around medication adherence and preparation for surgery were: (1) understanding illness; (2) understanding treatment; (3) role of health professional; (4) services and processes; (5) accessing hospital; (6) individual factors; (7) routines and habits. Supplemental Digital Content 2, Table S4, https://links.lww.com/AA/F794, summarizes the themes and subthemes.

#### Understanding Illness

This theme illustrates the sets of beliefs participants use to make sense of their illness. While some explanations aligned with medical views such as poor diet, sedentary lifestyle, and smoking (“Party, wrong eating… lack of exercise”) others reflected misperceptions, like attributing diabetes to a mining accident (“I was blown up underground in a goldmine. That shock gave me diabetes”). These beliefs often shaped participants’ rationales for managing their illness.

Several participants expressed strong emotional responses to living with comorbidities, often describing a sense of loss, restriction, and fear. One individual spoke of “loss of independence” and feeling “stranded” due to limited mobility and sensory loss, while others described concern over complications like slow healing or the fear of “losing feet and toes” if the condition were to become uncontrolled.

#### Understanding Treatment

This theme captures the complexity of beliefs surrounding medications used to manage chronic illness. Some participants expressed a strong sense of necessity (“If I don’t take my medication I know I won’t survive”) but also concerns about short-term harm such as hypoglycemia and long-term harm, such as kidney damage. This led some to alter doses or stop treatment altogether, reflecting a perceived trade-off: (“You’re damned if you do and damned if you don’t”). Medication decisions were also shaped by access issues and disruption of daily routine, with participants describing difficulty obtaining glucose monitors or synchronizing insulin with meals. Some participants viewed natural remedies as safer or more aligned with personal values, despite acknowledging their unpredictability (“With natural medicines, it’s trial and error… but it’s all nature inside”). As this study focused on beliefs about chronic illness and medications in patients preparing for surgery, discussion of surgery and surgical decision-making was not specifically included in the questions asked or participant accounts. The study was conducted at a single point along the care pathway, which limited exploration of how preparation for surgery and related information evolved over time.

#### Health Professional Role

Participants expressed a mix of trust, passivity, and frustration in their interactions with health professionals. Some viewed doctors as life-saving experts (“They are trying to save our lives”) and accepted treatment without question. Others expressed skepticism, noting rushed appointments and a lack of personalized care (“They’re very quick to get you out of the room… those who sit and talk to me, I trust far more”). Positive experiences were linked to clinicians who listened attentively, admitted uncertainty, or explained treatment decisions. In contrast, unexplained changes to medications, especially when participants felt stable, led to confusion, mistrust, and reduced adherence (“If something’s working, why change it?”).

#### Services, Processes, and Accessing Hospital

Participants described the considerable effort involved in navigating the healthcare system to obtain medication and attend appointments. Long waits at day hospitals (“six, seven hours just for medication”) and crowded conditions led some to return home. Others appreciated systems that prioritized vulnerable individuals or allowed for 2-month prescriptions, easing the burden of frequent visits. Transport, safety, and cost were major barriers, with some walking long distances or relying on borrowed taxi fares, while others expressed fear of crime en route to care facilities. Home delivery and community-based collection points were highly valued, offering safer, faster, and more convenient access to medicines (“They deliver it once a month… it’s in little boxes,” and “It’s nice and smooth… in and out”).

#### Individual Factors, Routines, and Habits

Participants described a wide range of personal factors influencing their ability to manage their illness. Reasons for medication adherence varied from fear of consequences, such as collapsing without tablets, to practical barriers such as pharmacy opening times or social distractions. While some expressed aversion to lifelong medication (“I’ll need to drink the tablets until I die”), others accepted it as necessary. Lifestyle changes were often made with the intention of improving health, such as cutting sugar or switching to lower-fat foods, but these were not always sustainable or affordable (“He gave me diet food…you can’t, it’s too expensive”). Maintaining behavioral changes was complicated by religious events, social settings, or unstructured daily routines, particularly after retirement (“When I was working, I had a schedule…now I sleep my life away”). Social support and routine emerged as key enablers of adherence. Participants described strategies such as pre-packing tablets, using visual cues, and relying on family reminders (“My daughter put my water…she knows it’s pill time”) to stay on track with their treatment.

## DISCUSSION

This study explored the experiences of noncardiac surgical patients with cardiovascular comorbidities in a South African public-sector setting, focusing on their beliefs about chronic illness, medication adherence, and involvement in treatment decisions. Using a mixed-methods design, we identified individual, relational, and system-level factors influencing how patients engage with chronic disease management in the perioperative setting. The findings demonstrate the feasibility of integrating brief self-report tools into anesthetic and surgical care pathways to elicit patient perspectives, even in resource-limited environments.

Participants generally found the questionnaires acceptable, although many required verbal administration. This underscores the need for adaptable approaches in multilingual and low-literacy settings common across low- and middle-income countries.^23^ The use of verbal facilitation and translation could be integrated into preoperative care where written forms are a barrier to effective assessment.

Findings from the BIPQ revealed that participants largely understood their illness as chronic and expressed confidence in treatment and self-management. However, high levels of concern and emotional distress were also reported, which is consistent with the reported high incidence of preoperative anxiety in low- and middle-income countries.^24^ These responses are particularly relevant in the perioperative setting, where anxiety and fear about surgery may add to existing psychological burdens.^25^ Addressing this requires an approach to preoperative optimization that includes psychological and social factors.

The BMQ revealed strong beliefs about medication necessity, yet concerns about long-term harm, dependency, and lack of understanding were widespread. Nearly half of the respondents described their medication as a “mystery,” highlighting a critical gap in patient education. In low- and middle-income countries, where high patient loads and short consultation times are common, opportunities to explain treatment regimens in accessible ways may be missed. For the anesthesiologist assessing patients preoperatively, this may manifest as incomplete medication histories, unintentional nonadherence, or confusion around perioperative medication management. These findings support the need for targeted medication reconciliation and counseling during the perioperative period.^26^

Patients’ experiences of shared decision-making were mixed. Although most felt the final decision was right for them, few recalled being offered treatment options or being asked about their preferences. Where provider time is constrained, and patient volumes are high, shared decision-making can be challenging but is increasingly recognized as a key aspect of perioperative practice.^27^ Improving preoperative communication to include a discussion of patient priorities and concerns, even briefly may help bridge this gap and improve satisfaction with care.

Qualitative findings provided valuable context. Participants described substantial challenges in accessing care, including long waits, travel costs, and personal safety concerns, particularly in areas with high crime rates. In this context, services such as community-based dispensing and home delivery were highly valued. These models, already used in HIV and chronic disease programs across sub-Saharan Africa, could be extended to support perioperative medication continuity, particularly for patients with cardiovascular disease, where interruptions in therapy (eg, antihypertensive, antiplatelet, or diabetic medications) may have significant intra- and postoperative implications.^28^

Cultural beliefs about medicines influenced adherence. Data from the South African Prospective Urban and Rural Epidemiological (PURE) study raised concerns about the safety, regulation, and interactions of traditional and prescribed medicines.^29^ The use of traditional medicines is associated with lower adherence in prescribed medicines, and in hypertensive patients, this leads to a higher blood pressure with more severe complications.^30,31^ Almost half of the participants believed natural remedies were safer than prescribed drugs, and some described family encouragement to use traditional treatments. Such beliefs may impact on perioperative care if patients withhold information about concurrent therapies or discontinue conventional treatment before surgery. Understanding patient beliefs and involving family members in preoperative discussions may improve concordance and safety.

Doctor–patient interactions were described ambivalently. Participants valued clinicians’ expertise but felt consultations were rushed and lacked opportunities for discussion. This finding is especially relevant to anesthesia, where preoperative assessments are often time-limited and episodic. Even a short, structured conversation that acknowledges patient perspectives may improve engagement and trust.

Our findings reflect known challenges related to pill burden and multimorbidity.^32^ Polypharmacy can lead to confusion and treatment fatigue particularly in settings where treatment regimens are not explained clearly or are frequently altered without patient understanding. Pharmacist involvement in simplification of regimens and routine medication review in preoperative clinics may help mitigate this.^33^ Ensuring medication availability, helping to create and support routines around taking medicines and providing reminders were found to be beneficial.^26^

Although the study explored patients’ beliefs using self-reported measures, no assumptions were made about the static or labile nature of participant’s beliefs about their illnesses. Increased engagement with services may influence their knowledge and views; however, this was not explored further in this preliminary study. This study has limitations. The sample was small and purposively selected. Objective measures of medication use, such as refill tracking or drug levels, were not feasible in this context. However, the integration of qualitative methods allowed us to understand not only what patients believed and did, but also why, offering critical insights for improving care.

Setting up the study highlighted the difficulty in identifying the most appropriate point along the care pathway at which to explore surgical decision-making and the provision of preparatory information by healthcare professionals to support behavior change. As a result, these elements were not examined systematically within this sample. Although the study aimed to explore experiences of preparing for noncardiac vascular surgery, the framing of the survey and interview questions may have shaped the focus of participants’ responses, and surgery-specific decision-making did not feature prominently. The limited discussion of surgery may therefore reflect both the emphasis of the interview questions and the possibility that shared decision-making for surgery is addressed inconsistently across hospital and outreach services. Further research focusing explicitly on surgical decision-making, using alternative question framing or targeted prompts, is required to explore this aspect in greater depth. Future work may also benefit from mapping or observing how preparatory surgical information is delivered by different healthcare professionals, alongside patient interviews.

For anesthesiologists and perioperative clinicians working in global health or low-resource settings, this study highlights the value of brief, structured, validated tools that can elicit important information about patient beliefs, barriers, and behaviors. Preoperative assessment in such settings should move beyond checklist-based risk stratification to encompass cultural competence, health literacy, and shared decision-making. While challenging in resource-limited settings, these approaches will likely be cost-effective by reducing short-term complications and long-term morbidity. They also have the potential to improve patient satisfaction. Future study should explore task-sharing models where community health workers support medication reconciliation, education, and follow-up. Next steps include research to more fully understand surgical decision-making, including targeted exploration of how preparatory information is communicated by healthcare professionals.

## DISCLOSURES

**Conflicts of Interest:** In the past 5 years, S. J. Howell has acted as a panel member for Edwards LifeSciences and is in receipt of research support from Abbott. No other authors declared Conflicts of Interest. **Funding:** The Thompson Trust travel scholarship awarded to Oluwanilo Awoloto - School of Medicine, University of Leeds (UOL), UK, in collaboration with Groote Schuur Hospital and the University of Cape Town (UCT), ZA. **This manuscript was handled by:** Tom Bashford, MBiochem, MBBS, PhD, MRCP, FRCA.

## ACKNOWLEDGMENTS

The authors thank Dr Nadraj Naidoo (Vascular Surgery Consultant) and Dr Kuda Katurura (Vascular Surgery Registrar), Groote Schuur Hospital, Cape Town, South Africa, and Dr Margot Flint, Principal Scientific Officer, Department of Anaesthesia and Perioperative Medicine, University of Cape Town, Cape Town, South Africa, who supported participant identification and data collection.

## Supplementary Material



## References

[1] Weiser TG Haynes AB Molina G. Estimate of the global volume of surgery in 2012: an assessment supporting improved health outcomes. Lancet. 2015;385(suppl 2):S11. 10.1016/S0140-6736(15)60806-6 26313057

[2] Dobson GP. Trauma of major surgery: a global problem that is not going away. Int J Surg. 2020;81:47–54. 10.1016/j.ijsu.2020.07.017 PMC7388795 32738546

[3] Biccard BM Madiba TE Kluyts HL; African Surgical Outcomes Study (ASOS) investigators. Perioperative patient outcomes in the African Surgical Outcomes Study: a 7-day prospective observational cohort study. Lancet. 2018;391:1589–1598. 10.1016/S0140-6736(18)30001-1 29306587

[4] Alphonsus CS Kluyts HL Gobin V. Towards the quantification of perioperative cardiovascular risk in the African context: a sub-analysis of the South African surgical outcomes study and the African surgical outcomes study. S Afr Med J. 2021;111:1065–1069. 10.7196/SAMJ.2021.v111i11.15848 34949270

[5] World Health Organization. Adherence to Long-Term Therapies: Evidence for Action. World Health Organization; 2003.

[6] Shin J Konlan KD. Prevalence and determinants of medication adherence among patients taking antihypertensive medications in Africa: a systematic review and meta-analysis 2010-2021. Nurs Open. 2023;10:3506–3518. 10.1002/nop2.1613 PMC10170912 36693022

[7] Croome N Ahluwalia M Hughes LD Abas M. Patient-reported barriers and facilitators to antiretroviral adherence in sub-Saharan Africa. AIDS. 2017;31:995–1007. 10.1097/QAD.0000000000001416 PMC5378008 28121707

[8] Okwuonu CG Ojimadu NE Okaka EI Akemokwe FM. Patient-related barriers to hypertension control in a Nigerian population. Int J Gen Med. 2014;7:345–353. 10.2147/IJGM.S63587 PMC4086668 25061335

[9] Abebe SM Berhane Y Worku A. Barriers to diabetes medication adherence in North West Ethiopia. Springerplus. 2014;3:195–195. 10.1186/2193-1801-3-195 PMC4021033 24834373

[10] Biccard BM Bandu R. Clinical risk predictors associated with cardiac mortality following vascular surgery in South African patients. Cardiovasc J Afr. 2007;18:216–220. PMC4170222 17940665

[11] Hagger MS Orbell S. The common sense model of illness self-regulation: a conceptual review and proposed extended model. Health Psychol Rev. 2022;16:347–377. 10.1080/17437199.2021.1878050 33461402

[12] Horne R Weinman J Hankins M. The beliefs about medicines questionnaire: the development and evaluation of a new method for assessing the cognitive representation of medication. Psychol Health. 1999;14:1–24.

[13] Broadbent E Wilkes C Koschwanez H Weinman J Norton S Petrie KJ. A systematic review and meta-analysis of the brief illness perception questionnaire. Psychol Health. 2015;30:1361–1385. 10.1080/08870446.2015.1070851 26181764

[14] Horne R Chapman SC Parham R Freemantle N Forbes A Cooper V. Understanding patients’ adherence-related beliefs about medicines prescribed for long-term conditions: a meta-analytic review of the necessity-concerns framework. PLoS One. 2013;8:e80633. 10.1371/journal.pone.0080633 PMC3846635 24312488

[15] LLC MMAR. Morisky Scales MMAS-4 & MMAS-8. 2025. Accessed 10 May 2025. Available at: https://www.moriskyscale.com/about-the-morisky-scale---mmas-4--mmas-8-the-morisky-scales.html

[16] Légaré F Kearing S Clay K. Are you SURE?: Assessing patient decisional conflict with a 4-item screening test. Can Fam Physician. 2010;56:e308–e314. PMC2920798 20705870

[17] Durand M-A Bekker HL Casula A. Can we routinely measure patient involvement in treatment decision-making in chronic kidney care? A service evaluation in 27 renal units in the UK. Clin Kidney J. 2016;9:252–259. 10.1093/ckj/sfw003 PMC4792631 26985377

[18] O’Cathain A Croot L Sworn K. Taxonomy of approaches to developing interventions to improve health: a systematic methods overview. Pilot Feasibility Stud. 2019;5:41. 10.1186/s40814-019-0425-6 PMC6419435 30923626

[19] O’Brien BC Harris IB Beckman TJ Reed DA Cook DA. Standards for reporting qualitative research: a synthesis of recommendations. Acad Med. 2014;89:1245–1251. 10.1097/ACM.0000000000000388 24979285

[20] Braun V Clarke V. To saturate or not to saturate? Questioning data saturation as a useful concept for thematic analysis and sample-size rationales. Qual Res Sport Exerc Health 2019;13:201–216.

[21] Braun V Clarke V. Using thematic analysis in psychology. Qual Res Psychol. 2006;3:77–101.

[22] Broadbent E Petrie KJ Main J Weinman J. The brief illness perception questionnaire. J Psychosom Res. 2006;60:631–637. 10.1016/j.jpsychores.2005.10.020 16731240

[23] Schlemmer A Mash B. The effects of a language barrier in a South African district hospital. S Afr Med J. 2006;96:1084–1087. 17164941

[24] Bedaso A Mekonnen N Duko B. Prevalence and factors associated with preoperative anxiety among patients undergoing surgery in low-income and middle-income countries: a systematic review and meta-analysis. BMJ Open. 2022;12:e058187. 10.1136/bmjopen-2021-058187 PMC8919464 35277412

[25] Fecher-Jones I Grimmett C Ainsworth B. Systematic review and narrative description of the outcomes of group preoperative education before elective major surgery. BJA Open. 2024;10:100286. 10.1016/j.bjao.2024.100286 PMC11145434 38832071

[26] Mahmood SBZ Rehman F Jamal A. Impact of a medication reconciliation improvement package on adherence to medication reconciliation among internal medicine physicians: a quality improvement project in a lower-middle income country. Hosp Pharm. 2024;59:624–630. 10.1177/00185787241253442 PMC11497527 39449857

[27] Shaw SE Hughes G Pearse R. Opportunities for shared decision-making about major surgery with high-risk patients: a multi-method qualitative study. Br J Anaesth. 2023;131:56–66. 10.1016/j.bja.2023.03.022 PMC10308437 37117099

[28] Hoke T Bateganya M Toyo O. How home delivery of antiretroviral drugs ensured uninterrupted HIV treatment during COVID-19: experiences from Indonesia, Laos, Nepal, and Nigeria. Glob Health Sci Pract. 2021;9:978–989. 10.9745/GHSP-D-21-00168 PMC8691873 34933991

[29] Hughes GD Aboyade OM Clark BL Puoane TR. The prevalence of traditional herbal medicine use among hypertensives living in South African communities. BMC Complement Altern Med. 2013;13:38. 10.1186/1472-6882-13-38 PMC3598715 23414344

[30] Schutte AE Srinivasapura Venkateshmurthy N Mohan S Prabhakaran D. Hypertension in low- and middle-income countries. Circ Res. 2021;128:808–826. 10.1161/CIRCRESAHA.120.318729 PMC8091106 33793340

[31] Lassale C Gaye B Diop IB. Use of traditional medicine and control of hypertension in 12 African countries. BMJ Glob Health. 2022;7:e008138. 10.1136/bmjgh-2021-008138 PMC9163537 35654446

[32] Nicholson K Liu W Fitzpatrick D. Prevalence of multimorbidity and polypharmacy among adults and older adults: a systematic review. Lancet Healthy Longev. 2024;5:e287–e296. 10.1016/S2666-7568(24)00007-2 38452787

[33] Porubcova S Szmicsekova K Lajtmanova K. Pharmacist-led interventions for vascular surgery patients: a prospective study on reducing drug-related problems. BMC Health Serv Res. 2024;24:1564. 10.1186/s12913-024-12015-7 PMC11653549 39695586

