# Supplementary material for: Patient Beliefs and Experiences of Adhering to Medical Therapies for Cardiovascular Comorbidities, Before Noncardiac Elective Surgery in South Africa: A Mixed-Methods Study
Source: Anesth Analg. 2026 Jul 3;143(6):1335–43. doi: 10.1213/ANE.0000000000008110 (PMC13747293; doi:10.1213/ANE.0000000000008110)
Supplement: Supplementary file 1 [file ane-143-1335-s001.pdf]

Full Title: Patient beliefs and experiences of adhering to medical therapies for cardiovascular comorbidities, before non-cardiac elective surgery in South Africa: a mixed-methods study.

Document S1: English version of survey

Patient experiences of medications for cardiovascular comorbidities before surgery: Questionnaire Survey

August 2022

Thank you for taking the time to answer this survey. Your answers are treated in confidence. All the responses we get from people in this clinic/ward will be summarised together. The doctors will not know the specific answers you give.

Your answers are important to the staff at this clinic. The findings will be used to improve how the clinic/ward can help patients be more involved with their treatment. If you are not sure of an answer, ask one of the research team for help. ***Oluwanilo Awoloto*** is the researcher who is carrying out this survey with Professor Bruce Biccard and Dr Christella Alphonsus.

This study is funded by an EXSEL travel scholarship awarded to Oluwanilo Awoloto - School of Medicine, University of Leeds (UK) in collaboration with Groote Schuur Hospital and the University of Cape Town (ZA).

Oluwanilo Awoloto is a third year medical student, supervised by Dr Simon Howell and Professor Hilary Bekker (UK), and Professor Bruce Biccard and Dr Christella Alphonsus (ZA).

STUDY NUMBER: ________________

DATE COMPLETED: ________________

SECTION 1

This part of the survey asks for details about you. Your answers help us to know who is using our clinic and what different people need when they have treatment here.

**Q1**	**What is your date of birth?**	_ _ (date) / _ _ _ _ _ _ _ _ _ _ (month) / _ _ _ _ (year)
**Q2**	**What is your marital status at this time (tick one answer)?**	☐ Single ☐ Married ☐ Divorced ☐Widowed<br>Other (list) ___________________________________
**Q3**	**Where do you live (tick one answer)?**	☐ City ☐ Township ☐ Rural<br>Other (list) __________________________
**Q4**	**How many people live in your home (list numbers)?**	Number of adults ____<br>Number of children ____<br>Other ______________________________
**Q5**	**Are you employed (tick one answer)?**	☐ Yes ☐ No
**Q6**	**What languages do you speak (tick all answer)?**	☐ Afrikaans ☐ English ☐ Xhosa<br>Other (list) __________________________________
**Q7**	**What is your highest education level (tick one answer)**	☐ No formal education ☐ Matric<br>☐ Professional Training ☐ Degree<br>Other (list) ____________________________________
**Q8**	**What answer best describes your religion (tick one answer)**	☐ Christianity ☐ Judaism<br>☐ Traditional African<br>☐ Hindu ☐ Sikh ☐ Islam<br>☐ No formal religion<br>Other (list) _________________________________
**Q9**	**Who pays for your health care (tick one answer)?**	☐ Me and My Family ☐ My Employer<br>☐ The Government ☐ Medical Aid<br>Other (list) _________________________________

SECTION 2

This section asks for details about the medications you are taking, the health problems you have, and some of your health habits. Please answer all questions.

**Q10**	**How long have you been sick for?**	Years_______ Months _________
**Q11**	**Do you have any allergies?**	☐ No<br>☐ Yes (list)____________________________
**Q12**	**List all the medications you are prescribed:**	_______________________________________<br>_______________________________________<br>_______________________________________<br>_______________________________________
**Q13**	**List any other health tonics or remedies you are taking:**	_______________________________________<br>_______________________________________<br>_______________________________________
**Q14**	**Did you bring all the medications you were prescribed to the hospital when you came?**	Yes☐ Some of them ☐ No☐
**Q15**	**Do you understand why you are taking your medication? (Please circle)**	0 1 2 3 4 5<br>Not at all … Very much
**Q16**	**Do you ever forget to take your medications? (Please circle)**	0 1 2 3 4 5<br>Not at all … Very much
**Q17**	**Do you know the benefits of the medications you are taking?**	Yes☐ No☐
**Q18**	**Do you know the risks of the medications you are taking?**	Yes☐ No☐
**Q19**	**Do any of your medications give you side effects? (Please circle)**	0 1 2 3 4 5<br>Not at all … Very much

**Q20**	**Which of these health problems do you have (tick all you say yes to)?**	☐ High blood pressure<br>☐ Heart failure<br>☐ Diabetes	☐ High cholesterol<br>☐ Heart disease<br>☐ Kidney disease

		☐ Peripheral vascular disease<br>Other (list) ________________________________
**Q21**	**Do any of these health problems run in the family (tick all you say yes to)?**	☐ High blood pressure ☐ High cholesterol<br>☐ Heart failure ☐ Heart disease<br>☐ Diabetes ☐ Kidney disease<br>☐ Peripheral vascular disease<br>Other (list) ________________________________
**Q22**	**Which of the health problems you have, most concerns you (tick one answer only)?**	☐ High blood pressure ☐ High cholesterol<br>☐ Heart failure ☐ Heart disease<br>☐ Diabetes ☐ Kidney disease<br>☐ Peripheral vascular disease<br>Other (list) ________________________________
**Q23**	**Have you had any surgery for any of your health problems?**	Yes☐ No☐

**Q24**	**What is your smoking status?**	☐ Never smoked<br>☐ Gave up smoking ___ years ____ months ago<br>☐ Smoke every now and then<br>☐ Smoke everyday<br>Other (list) __________________________________
**Q25**	**How much alcohol do you drink?**	☐ Do not drink alcohol<br>☐ Drink alcohol 3 or less days a week<br>☐ Drink alcohol more than 4 times a week<br>☐ Drink alcohol every day<br>Other (list) ____________________________________
**Q26**	**Do you take any substances to get high, to feel elevated, to get a buzz, to change mood or for pain?**	Yes☐<br>No☐

SECTION 3

The following questions are based on the one illness or health problem that most concerns you from Q24. For example, if the illness that most concerns you is diabetes – please answer these questions based on the medication you take for your diabetes. There are no right answers. It is your experiences that matter.
Please circle the number that best corresponds to your views:

**Q27**	**How much does your illness affect your life?**	0 1 2 3 4 5 6 7 8 9 10<br>No affect at all ... Severely affects my life
**Q28**	**How long do you think your illness will continue?**	0 1 2 3 4 5 6 7 8 9 10<br>A very short time ... Forever
**Q29**	**How much control do you feel you have over your illness**	0 1 2 3 4 5 6 7 8 9 10<br>Absolutely no control ... Extreme amount of control
**Q30**	**How much do you think your treatment can help your illness?**	0 1 2 3 4 5 6 7 8 9 10<br>Not at all ... Extremely helpful
**Q31**	**How much do you experience symptoms from your illness?**	0 1 2 3 4 5 6 7 8 9 10<br>No symptoms at all ... Many severe symptoms
**Q32**	**How concerned are you about your illness?**	0 1 2 3 4 5 6 7 8 9 10<br>Not at all concerned ... Extremely concerned
**Q33**	**How much do you feel you understand your illness?**	0 1 2 3 4 5 6 7 8 9 10<br>Don't understand at all ... Understand very clearly
**Q34**	**How much does your illness affect you emotionally? (e.g. does it make you angry, scared, upset or depressed?)**	0 1 2 3 4 5 6 7 8 9 10<br>Not at all affected emotionally ... Extremely affected emotionally
**Q35**	**List in rank-order the three most important factors that you believe caused your illness.**	The most important causes for me are:<br>1. ____________________<br>2. ____________________<br>3. ____________________

<u>SECTION 4</u>

This section has statements that other people have made about medicines.
Please answer these questions based on the <u>one</u> health problem that most concerns you from Q24.
For example, if the illness that most concerns you is diabetes – please answer these questions based on the medication you take for your diabetes There are no right or wrong answers.
It is your experiences that matter.
Please tick one box for each item that best matches your personal view:

**Q36**	**My health at present depends on my medication**	Strongly agree ☐	Agree ☐	Uncertain ☐	Disagree ☐	Strongly disagree ☐
**Q37**	**Having to take medication worries me**	Strongly agree ☐	Agree ☐	Uncertain ☐	Disagree ☐	Strongly disagree ☐
**Q38**	**My life would be impossible without my medication**	Strongly agree ☐	Agree ☐	Uncertain ☐	Disagree ☐	Strongly disagree ☐
**Q39**	**Without my medication I would be very ill**	Strongly agree ☐	Agree ☐	Uncertain ☐	Disagree ☐	Strongly disagree ☐
**Q40**	**I sometimes worry about the long-term effects of my medication**	Strongly agree ☐	Agree ☐	Uncertain ☐	Disagree ☐	Strongly disagree ☐
**Q41**	**My medication is a mystery to me**	Strongly agree ☐	Agree ☐	Uncertain ☐	Disagree ☐	Strongly disagree ☐
**Q42**	**My health in the future will depend on my medication**	Strongly agree ☐	Agree ☐	Uncertain ☐	Disagree ☐	Strongly disagree ☐
**Q43**	**My medication disrupts my life**	Strongly agree ☐	Agree ☐	Uncertain ☐	Disagree ☐	Strongly disagree ☐
**Q44**	**I sometimes worry about becoming too dependent on my medication**	Strongly agree ☐	Agree ☐	Uncertain ☐	Disagree ☐	Strongly disagree ☐
**Q45**	**My medication protects me from becoming worse**	Strongly agree ☐	Agree ☐	Uncertain ☐	Disagree ☐	Strongly disagree ☐

The following questions are about your personal views about medicines in general.

These are statements other people have made about medicines in general.

Please tick one box for each item that best matches your view. There are no right or wrong answers.

**Q46**	**Doctors use too many medicines**	Strongly agree ☐	Agree ☐	Uncertain ☐	Disagree ☐	Strongly disagree ☐
**Q47**	**People who take medicines should stop their treatment for a while every now and again.**	Strongly agree ☐	Agree ☐	Uncertain ☐	Disagree ☐	Strongly disagree ☐
**Q48**	**Most medicines are addictive.**	Strongly agree ☐	Agree ☐	Uncertain ☐	Disagree ☐	Strongly disagree ☐
**Q49**	**Natural remedies are safer than medicines**	Strongly agree ☐	Agree ☐	Uncertain ☐	Disagree ☐	Strongly disagree ☐
**Q50**	**Medicines do more harm than good.**	Strongly agree ☐	Agree ☐	Uncertain ☐	Disagree ☐	Strongly disagree ☐
**Q51**	**All medicines are poisons**	Strongly agree ☐	Agree ☐	Uncertain ☐	Disagree ☐	Strongly disagree ☐
**Q52**	**Doctors place too much trust on medicines**	Strongly agree ☐	Agree ☐	Uncertain ☐	Disagree ☐	Strongly disagree ☐
**Q53**	**If doctors had more time with patients, they would prescribe fewer medicines.**	Strongly agree ☐	Agree ☐	Uncertain ☐	Disagree ☐	Strongly disagree ☐

SECTION 5.

This section asks for your views about making treatment decisions with your doctor for the health problem that most concerns you from Q24. For example, if the illness that most concerns you is diabetes – please answer these questions based on the medication you take for your diabetes.
What was your experience of making this treatment decision with your doctor?
For each statement, tick the box that matches best how much you agree with it:

		Agree Strongly	Agree	Not Sure	Disagree	Disagree Strongly
	The doctor…					
Q54	talked about other options from the one I chose	☐	☐	☐	☐	☐
Q55	talked about whether or not there was a medically best option for my health problem	☐	☐	☐	☐	☐
Q56	gave their views about the options	☐	☐	☐	☐	☐
Q57	asked for my views about the options	☐	☐	☐	☐	☐
	I talked about…					
Q58	what was important to me about this decision	☐	☐	☐	☐	☐
Q59	why one option suited me better than another	☐	☐	☐	☐	☐
Q60	the risks and benefits of the options for me and my health	☐	☐	☐	☐	☐
	I felt…					
Q61	it would be OK to choose any option we talked about	☐	☐	☐	☐	☐
Q62	the decision made was the best one for me	☐	☐	☐	☐	☐
Q63	the doctor and I agreed which option was the best one for me	☐	☐	☐	☐	☐

Please tick this box if you are happy to have a follow-up interview based on the answers you have provided above ☐

Thank you for taking part in this survey.
Your answers will be used by the clinical team here to improve their services.

Document S2: Afrikaans version of Survey

Pasiënt ervarings van medikasie vir kardiovaskulêre komorbiditeite voor die operasie: Vraelys Opname

Augustus 2022

Dankie dat jy die tyd geneem het om hierdie opname te beantwoord. Ons antwoorde word met vertroue behandel. Al die antwoorde wat ons van mense in hierdie kliniek /saal kry, sal saam opgesom word. Die dokters sal nie die spesifieke antwoorde ken wat u beantwoord nie.

U antwoorde is belangrik vir die personeel by hierdie kliniek/saal. Die bevindinge sal gebruik word om te verbeter hoe die kliniek / saal pasiënte kan help om meer betrokke te wees by hul behandeling. As jy nie seker is van 'n antwoord nie, vra een van die navorsing span vir hulp. ***Oluwanilo Awoloto*** is die navorser wat hierdie opname saam met Professor Bruce Biccard en Dr Christella Alphonsus uitvoer.

Hierdie studie word befonds deur 'n EXSEL-reisbeurs wat aan Oluwanilo Awoloto - Skool vir Geneeskunde, Universiteit van Leeds (VK) in samewerking met die Groote Schuur hospitaal en die Universiteit van Kaapstad (UCT), (RSA) toegeken is.

Oluwanilo Awoloto is 'n derde jaar mediese student, onder toesig van Dr Simon Howell en Professor Hilary Bekker (VK), en Professor Bruce Biccard en Dr Christella Alphonsus (RSA).

STUDIE NOMMER: ___________

DATUM VOLTOOI: ___________

AFDELING 1

Hierdie deel van die opname vra vir besonderhede oor jou. U antwoorde help ons om te weet wie ons kliniek/saal gebruik en wat verskillende mense benodig wanneer hulle hier behandeling ontvang.

**Q1**	**Wat is jou geboortedatum?**	_ _ (datum) / _ _ _ _ _ _ _ _ _ _ _ _(maand) / _ _ _ _ _ _(jaar)
**Q2**	**Wat is jou huwelik status op hierdie tydstip (merk een antwoord)?**	☐ Ongetroude ☐ getroude ☐ weduwee ☐ Ander (lys) __
**Q3**	**Waar woon jy (merk een antwoord)?**	☐ Stad ☐ Dorp ☐ Landelike<br>Ander (lys) _____
**Q4**	**Hoeveel mense woon in jou huishouding (lys nommers)?**	Aantal volwassenes ____<br>Aantal kinders ____<br>Ander ______
**Q5**	**Werk jy (merk een antwoord)?**	☐ Ja ☐ Nee
**Q6**	**Watter tale praat jy (merk alle antwoord)?**	☐ Afrikaans ☐ Engels ☐ Xhosa<br>Ander (lys) __
**Q7**	**Wat is jou hoogste kwalifikasie (merk een antwoord)**	☐ Geen formele onderwys ☐ Matriek<br>☐ Professionele Opleidingsgraad ☐<br>Ander (lys) __
**Q8**	**Watter antwoord beskryf jou godsdiens die beste (merk een antwoord)**	☐ Christendom ☐ Judaïsme<br>☐ Tradisionele Afrikaans<br>☐ Hindoe ☐ Sikh ☐ Islam<br>☐ Geen formele godsdiens nie<br>Ander (lys) _____
**Q9**	**Wie betaal vir jou gesondheidsorg (merk een antwoord)?**	☐ Ek en my familie ☐ my werkgewer<br>☐ Mediese Fonds van die Regering ☐<br>Ander (lys) ________________________

AFDELING 2

Hierdie afdeling vra vir besonderhede oor die medikasie wat jy gebruik, die gesondheids probleme wat jy het, en sommige van jou gesondheids gewoontes. Beantwoord asseblief alle vrae.

**Q10**	**Hoe lank is jy al siek?**	Years_______ maande _________
**Q11**	**Het jy enige allergieë?**	☐ Nee<br>☐ Ja (list)____________________
**Q12**	**Lys al die medikasie wat u voorgeskryf word:**	____________________________________<br>____________________________________<br>_____________________________________<br>___________________________________
**Q13**	**Lys enige ander gesondheids tonikums of middels wat jy neem:**	____________________________________<br>____________________________________<br>_____________________________________
**Q14**	**Het jy al die medikasie wat jy voorgeskryf is, saam na die hospitaal gebring?**	Sommige☐ van hulle ☐ nee☐
**Q15**	**Verstaan jy waarom jy jou medikasie neem? (Sirkel asseblief)**	0 1 2 3 4 5<br>Glad nie ... Baie baie
**Q16**	**Het jy al ooit vergeet om jou medikasie te neem? (Sirkel asseblief)**	0 1 2 3 4 5<br>Glad nie ... Baie baie
**Q17**	**Ken jy die voordele van die medikasie wat jy neem?**	Ja☐ Nee☐
**Q18**	**Ken jy die risiko's van die medikasie wat jy neem?**	Ja☐ Nee☐
**Q19**	**Gee enige van jou medikasie wat jy gebruik jou enige newe-effekte? (Sirkel asseblief)**	0 1 2 3 4 5<br>Glad nie ... Baie baie

Q20	**Watter van hierdie gesondheids probleme het jy (merk alles waarvoor jy ja sê)?**	☐ High bloeddruk ☐ Hoë cholesterol<br>☐ Hartversaking ☐ Hartsiektes<br>☐ Diabetes ☐ Niersiekte<br>☐ Peripherale vaskulêre siekte ☐Longsiekte<br>Other (lys) __
Q21	**Is daar enige gesondheids probleme in die familie (merk al you sê ja om)?**	☐ High bloeddruk ☐ Hoë cholesterol<br>☐ Hartversaking ☐ Hartsiektes<br>☐ Diabetes ☐ Niersiekte<br>☐ Peripherale vaskulêre siekte ☐Longsiekte<br>Other (lys) __
Q22	**Watter van die gesondheids probleme wat jy het, is jy die meeste bekommerd oor (merk slegs een antwoord)?**	☐ High bloeddruk ☐ Hoë cholesterol<br>☐ Hartversaking ☐ Hartsiektes<br>☐ Diabetes ☐ Niersiekte<br>☐ Peripherale vaskulêre siekte ☐Longsiekte<br>Other (lys) __
Q23	**Het u 'n operasie vir enige van u gesondheids probleme gehad?**	Ja☐ Nee☐

Q24	**Wat is u rook status?**	☐ Rook nie<br>☐ Het opgehou rook ___ jare ____ maande gelede<br>☐ Rook elke nou en dan<br>☐ Rook elke dag<br>Ander (lys) __
Q25	**Hoeveel alkoholiese drankies geniet u?**	☐ Moenie alkohol drink nie<br>☐ Drink alkohol 3 of less dae per week<br>☐ Drink alkohol meer as 4 keer per week<br>☐ Drink alkohol elke dag<br>Ander (lys) __
Q26	**Neem jy enige dwelm middels om hoog te word, om verhef te voel, of om 'n gons te kry, om u bui of pyn te verander?**	Ja☐<br>Nee☐

AFDELING 3

Die volgende vrae is gebaseer op die een siekte of gesondheidsprobleem wat u die meeste van V24 bekommer. By woorbeeld – As die siekte wat jy die meerste bekomer, diabetes is antwoord asseblief wierdie vrae gebaseer op die medikasie wat jy vir diabetes vatDaar is geen regte antwoorde nie. Dit is jou ervaring wat saak maak.

Sirkel asseblief die nommer wat die beste ooreenstem met jou sienings:

**Q27**	**Wtter impak het jou siekte op jou lewe?**	0 1 2 3 4 5 6 7 8 9 10<br>Geen invloed nie … Dit het 'n groot invloed op my lewe
**Q28**	**Hoe lank dink jy sal jou siekte voort duur?**	0 1 2 3 4 5 6 7 8 9 10<br>'N Baie kort tyd … Altyd
**Q29**	**Hoeveel beheer voel jy het jy oor jou siekte?**	0 1 2 3 4 5 6 7 8 9 10<br>Absoluut geen beheer nie … Uiterste hoeveelheid beheer
**Q30**	**Dink jy dat behandeling jou siekte kan verlig of help?**	0 1 2 3 4 5 6 7 8 9 10<br>Glad nie … Uiters nuttig
**Q31**	**Hoeveel keer ervaar jy simptome van jou siekte?**	0 1 2 3 4 5 6 7 8 9 10<br>Geen simptome nie … Baie ernstige simptome
**Q32**	**Hoe bekommerd is jy oor jou siekte?**	0 1 2 3 4 5 6 7 8 9 10<br>Glad nie bekommerd nie … Uiters bekommerd
**Q33**	**Voel jy dat jy jou siekte enig sins verstaan?**	0 1 2 3 4 5 6 7 8 9 10<br>Verstaan glad nie … Verstaan baie duidelik
**Q34**	**Raak jou siekte jou emosioneel aan? (Bv. maak dit jou kwaad, bang, ontsteld of depressief?)**	0 1 2 3 4 5 6 7 8 9 10<br>Glad nie emosioneel beïnvloed nie … Uiters geraak emosioneel
**Q35**	**Lys in rangorde die drie belangrikste faktore wat jy glo jou siekte veroorsaak het.**	Die belangrikste oorsake vir my is:<br>1. ______________________<br>2. ______________________<br>3. ______________________

<u>AFDELING 4</u>

Hierdie afdeling het verklarings wat ander mense oor medisyne gemaak het.
Beantwoord asseblief hierdie vrae gebaseer op die een gesondheids probleme wat die meeste van V24 bekommer het. By woorbeeld – As die siekte wat jy die meerste bekomer, diabetes is antwoord asseblief wierdie vrae gebaseer op die medikasie wat jy vir diabetes vat. Daar is geen regte of verkeerde antwoorde nie. Dit is jou ervaring wat saak maak.
Merk asseblief een boks vir elke antwoord wat die beste by u persoonlike mening/opininie pas:

Q36	**My gesondheid hang tans af van my medikasie af.**	Stem sterk saam ☐	Saamstem ☐	Onseker ☐	Verskil ☐	Sterk verskil ☐
Q37	**Om medikasie te neem, bekommer my.**	Stem sterk saam ☐	Saamstem ☐	Onseker ☐	Verskil ☐	Sterk verskil ☐
Q38	**My lewe sou onmoontlik wees sonder my medikasie.**	Stem sterk saam ☐	Saamstem ☐	Onseker ☐	Verskil ☐	Sterk verskil ☐
Q39	**Sonder my medikasie sal ek baie siek wees**	Stem sterk saam ☐	Saamstem ☐	Onseker ☐	Verskil ☐	Sterk verskil ☐
Q40	**Ek is soms bekommerd oor die langtermyn- newe effekte van my medikasie**	Stem sterk saam ☐	Saamstem ☐	Onseker ☐	Verskil ☐	Sterk verskil ☐
Q41	**My medikasie is 'n raaisel/ misterie vir my.**	Stem sterk saam ☐	Saamstem ☐	Onseker ☐	Verskil ☐	Sterk verskil ☐
Q42	**My gesondheid in die toekoms sal van my medikasie afhang.**	Stem sterk saam ☐	Saamstem ☐	Onseker ☐	Verskil ☐	Sterk verskil ☐
Q43	**My medikasie ontwrig my lewe**	Stem sterk saam ☐	Saamstem ☐	Onseker ☐	Verskil ☐	Sterk verskil ☐
Q44	**Ek is soms bekommerd dat ek te afhanklik van my medikasie raak**	Stem sterk saam ☐	Saamstem ☐	Onseker ☐	Verskil ☐	Sterk verskil ☐
Q45	**My medikasie beskerm my om erger/ sieker te word**	Stem sterk saam ☐	Saamstem ☐	Onseker ☐	Verskil ☐	Sterk verskil ☐

Die volgende vrae gaan oor jou persoonlike sienings/ opinie oor medisyne in die algemeen.

Dit is stellings wat ander mense oor medisyne in die algemeen gemaak het.

Merk asseblief een boks vir elke item wat die beste by jou aansig pas. Daar is geen regte of verkeerde antwoorde nie.

**Q46**	**Dokters gebruik te veel medisyne**	Stem sterk saam ☐	Saam stem ☐	Onseker ☐	Verskil ☐	Sterk verskil ☐
**Q47**	**Mense wat medisyne neem, moet hul medikasie elke nou en dan vir 'n rukkie stop.**	Stem sterk saam ☐	Saam stem ☐	Onseker ☐	Verskil ☐	Sterk verskil ☐
**Q48**	**Die meeste medisyne is verslawend.**	Stem sterk saam ☐	Saam stem ☐	Onseker ☐	Verskil ☐	Sterk verskil ☐
**Q49**	**Natuurlike middels is veiliger as medisyne**	Stem sterk saam ☐	Saam stem ☐	Onseker ☐	Verskil ☐	Sterk verskil ☐
**Q50**	**Medisyne doen meer skade as goed.**	Stem sterk saam ☐	Saam stem ☐	Onseker ☐	Verskil ☐	Sterk verskil ☐
**Q51**	**Alle medisyne is gifstowwe**	Stem sterk saam ☐	Saam stem ☐	Onseker ☐	Verskil ☐	Sterk verskil ☐
**Q52**	**Dokters plaas te veel vertroue in medisyne**	Stem sterk saam ☐	Saam stem ☐	Onseker ☐	Verskil ☐	Sterk verskil ☐
**Q53**	**As dokters meer tyd met pasiënte deur bring, sou hulle dalk minder medisyne voorskryf.**	Stem sterk saam ☐	Saam stem ☐	Onseker ☐	Verskil ☐	Sterk verskil ☐

AFDELING 5.

Hierdie afdeling vra u mening van behandelings besluite met u dokter vir die gesondheids probleem wat u die meeste van V24 bekommer. By woorbeeld – As die siekte wat jy die meerste bekomer, diabetes is antwoord asseblief wierdie vrae gebaseer op die medikasie wat jy vir diabetes vat.
Wat was jou ervaring om hierdie behandeling sbesluit met jou dokter te neem?
Vir elke stelling, merk die blokkie wat ooreenstem met die beste hoeveel jy daarmee saamstem:

		Stem sterk saam	Saamstem	Nie seker nie	Verskil	Verskil sterk
	Die dokter…					
Q54	gepraat oor ander opsies van die een wat ek gekies het	☐	☐	☐	☐	☐
Q55	gepraat of daar 'n beste mediese opsie vir my gesondheids probleem is of nie	☐	☐	☐	☐	☐
Q56	het hul sienings/ mening oor die opsies gegee	☐	☐	☐	☐	☐
Q57	gevra vir my sienings/ mening oor die opsies	☐	☐	☐	☐	☐
	Ek het gepraat oor…					
Q58	Wat was vir my belangrik oor hierdie besluit?	☐	☐	☐	☐	☐
Q59	waarom een opsie my beter as 'n ander pas	☐	☐	☐	☐	☐
Q60	die risiko's en voordele van die opsies vir my en my gesondheid	☐	☐	☐	☐	☐
	Ek het gevoel…					
Q61	dit sal in order/ OK wees om enige opsie te kies waaroor ons gepraat het	☐	☐	☐	☐	☐
Q62	Die besluit wat geneem is, was die beste een vir my	☐	☐	☐	☐	☐
Q63	die dokter en ek het ingestem watter opsie die beste een vir my was	☐	☐	☐	☐	☐

Merk asseblief hierdie blokkie as u gelukkig is om 'n opvolg onderhoud te hê op grond van die antwoorde wat u hierbo ☐

Dankie dat jy aan hierdie opname deelgeneem het.
U antwoorde sal hier deur die kliniese/ saal span gebruik word om hul dienste te verbeter.

Document S3: Isi-Xhosa version of Survey

Amava ezigulane zamayeza e-cardiovascular comorbidities ngaphambi kokuhlinzwa: UPhando lwemibuzo

EyeThupha 2022

Enkosi ngokuthatha ixesha lokuphendula olu phando. Iimpendulo zakho ziphathwa ngokuzithemba. Zonke iimpendulo esizifumana kubantu bale kliniki/wadi ziya kushwankathelwa kunye. Oogqirha abayi kuzazi iimpendulo ezithile ozinikayo.

Iimpendulo zakho zibalulekile kubasebenzi bale kliniki. Iziphumo ziya kusetyenziselwa ukuphucula indlela ikliniki/iwadi enokunceda ngayo izigulane ukuba zibandakanyeke ngakumbi kunyango lwazo. Ukuba akuqinisekanga ngempendulo, cela uncedo komnye weqela lophando. ***U-Oluwanilo Awoloto*** ngumphandi owenza olu phando kunye noNjingalwazi Bruce Biccard kunye noGqr Christella Alphonsus.

Olu phononongo luxhaswa ngemali ye-EXSEL yokufunda yohambo eyanikezelwa ku-Oluwanilo Awoloto - School of Medicine, University of Leeds (UK) ngokubambisana neSibhedlele saseGroote Schuur kunye neYunivesithi yaseKapa (ZA).

U-Oluwanilo Awoloto ungumfundi wezonyango owenza unyaka wesithathu, ephantsi kweliso likaGqr Simon Howell kunye noNjingalwazi uHilary Bekker (e-UK), kunye noNjingalwazi Bruce Biccard kunye noGq Christella Alphonsus (ZA).

INOMBOLO YOKUFUNDA: ________________

UMHLA OQEDIWEYO: _________________

ICANDELO 1

Le nxalenye yophando ibuza iinkcukacha ngawe. Iimpendulo zakho zisinceda sazi ukuba ngubani osebenzisa ikliniki yethu kwaye yintoni efunwa ngabantu abohlukeneyo xa benonyango apha.

**Q1**	**Luthini usuku lwakho lokuzalwa?**	_ _ (umhla) / _ _ _ _ _ _ _ _ _ (inyanga) / _ _ _ _ (unyaka)
**Q2**	**Sithini isimo sakho somtshato ngeli xesha (phawula impendulo enye)?**	☐ Awutshatanga ☐ Utshatile ☐ Uqhawule umtshato ☐ Umhlolokazi<br><br>Okunye (uluhlu) ______________________________
**Q3**	**Uhlala phi (phawula impendulo enye)?**	☐ Isixeko ☐ Ilokishi ☐ Emaphandleni<br>Okunye (uluhlu) ______________________
**Q4**	**Bangaphi abantu abahlala kwikhaya lakho (dwelisa amanani)?**	Inani labantu abadala _____<br>Inani labantwana _____<br>Okunye __________________________________
**Q5**	**Ngaba uyasebenza (phawula impendulo ibe nye)?**	☐ Ewe ☐ Hayi
**Q6**	**Ziziphi iilwimi ozithethayo (phawula zonke iimpendulo)?**	☐ IsiBhulu ☐ IsiNgesi ☐ IsiXhosa<br><br>Okunye (uluhlu) _____________________________
**Q7**	**Leliphi inqanaba lakho eliphezulu lemfundo (phawula impendulo enye)**	☐ Akukho mfundo isesikweni ☐ Imatriki<br>☐ Uqeqesho lobuNgcali ☐ Isidanga<br><br>Okunye (uluhlu) ______________________________
**Q8**	**Yeyiphi impendulo eyichaza ngcono inkolo yakho (phawula impendulo enye)**	☐ UbuKristu ☐ UbuYuda<br><br>☐ UmNtu waseAfrika<br><br>☐ UmHindu ☐ isiSikh ☐ iSilamsi<br><br>☐ Akukho nkolo isemthethweni<br>Olunye (uluhlu)<br><br>_____________________________________
**Q9**	**Ngubani ohlawulela ukhathalelo lwempilo yakho (phawula impendulo enye)?**	☐ Mna noSapho Lwam ☐ Umqeshi Wam<br><br>☐ Urhulumente ☐ Unyango Uncedo<br><br>Olunye (uluhlu)<br><br>_____________________________________

ICANDELO 2

Eli candelo libuza iinkcukacha malunga namayeza owasebenzisayo , iingxaki zempilo onazo, kunye neminye imikhwa yakho yempilo. Nceda uphendule yonke imibuzo.

**Q10**	**Unexesha elingakanani ugula?**	Iminyaka_____ Iinyanga _________
**Q11**	**Ingaba kukho nakuphi na ukwaliwa kwakho?**	☐ Hayi<br>☐ Ewe<br>(uluhlu)_____________________________________
**Q12**	**Dwelisa onke amayeza owamiselweyo:**	____<br>____<br>____<br>_______________________________________
**Q13**	**Dwelisa naziphi na ezinye iitoni zempilo okanye amayeza owathathayo:**	____<br>____<br>____
**Q14**	**Uze nawo onke amayeza ubuwanikwe esibhedlele xa usiza?**	Ewe ☐ Abanye babo ☐ Hayi ☐
**Q15**	**Ngaba uyasiqonda isizathu sokuba uthabathe iyeza lakho? (Nceda wenze isangqa)**	0 1 2 3 4 5<br>Unotshe Kakhulu kakhulu
**Q16**	**Ngaba ukhe ulibale ukuthatha amayeza akho? (Nceda wenze isangqa)**	0 1 2 3 4 5<br>Unotshe Kakhulu kakhulu
**Q17**	**Ngaba uyazazi iinzuzo zamayeza owathathayo?**	Ewe ☐ Hayi ☐
**Q18**	**Ngaba uyazazi iingozi zamayeza owathathayo?**	Ewe ☐ Hayi ☐
**Q19**	**Ngaba nawaphi na amayeza akho akunika iziphumo ebezingalindelekanga? (Nceda wenze isangqa)**	0 1 2 3 4 5<br>Unotshe Kakhulu kakhulu

**Q20**	**Zeziphi kwezi ngxaki zempilo onazo (phawula kuzo zonke onokuthi ewe kuzo)?**	☐ Uxinzelelo lwegazi oluphezulu ☐ I-cholesterol ephezulu<br>☐ Ukungaphumeleli kwentliziyo ☐ Isifo senhliziyo<br>☐ Isifo seswekile ☐ Isifo sezintso<br>☐ I-peripheral vascular disease ☐ Isifo semiphunga<br>Okunye (uluhlu) ________________________________
**Q21**	**Ngaba kukho nayiphi na kwezi ngxaki zempilo eziqhuba kusapho (phawula kuzo zonke onokuthi ewe kuzo)?**	☐ Uxinzelelo lwegazi oluphezulu ☐ I-cholesterol ephezulu<br>☐ Ukungaphumeleli kwentliziyo ☐ Isifo senhliziyo<br>☐ Isifo seswekile ☐ Isifo sezintso<br>☐ I-peripheral vascular disease ☐ Isifo semiphunga<br>Okunye (uluhlu) ________________________________
**Q22**	**Zeziphi iingxaki zempilo onazo, ezikukhathaza kakhulu (phawula impendulo enye kuphela)?**	☐ Uxinzelelo lwegazi oluphezulu ☐ I-cholesterol ephezulu<br>☐ Ukungaphumeleli kwentliziyo ☐ Isifo senhliziyo<br>☐ Isifo seswekile ☐ Isifo sezintso<br>☐ I-peripheral vascular disease ☐ Isifo semiphunga<br>Okunye (uluhlu) ________________________________
**Q23**	**Ngaba ukhe wenza utyando ngenxa yazo naziphi na iingxaki zempilo?**	Ewe ☐ Hayi ☐

**Q24**	**Sithini isimo sakho sokutshaya?**	☐ Zange utshaye<br>☐ Uyekile ukutshaya ___ iminyaka ____ kwiinyanga ezidlulileyo<br>☐ Tshaya qho<br>☐ Tshaya yonke imihla<br>Okunye (uluhlu) __________________________________
**Q25**	**Usela utywala obungakanani?**	☐ Musa ukusela utywala<br>☐ Sela utywala iintsuku ezi-3 okanye ngaphantsi ngeveki<br>☐ Sela utywala ngaphezu kwamaxesha ama-4 ngeveki<br>☐ Sela utywala yonke imihla<br>Okunye (uluhlu)<br>____

**Q26**	**Ngaba uthatha naziphi na izinto ukuze unyuke, uzive uphakamile, ukufumana i-buzz, ukutshintsha imo okanye intlungu?**	Ewe ☐<br>Hayi ☐

ICANDELO 3

Le mibuzo ilandelayo isekelwe kwisigulo esinye okanye ingxaki yempilo ekukhathazayo kakhulu ukusuka kwi-Q24. Akukho zimpendulo zichanekileyo. Ngamava akho abalulekileyo.
Nceda wenze isangqa kwinombolo engqinelana kakuhle nezimvo zakho:

**Q27**	**Isigulo sakho sibuchaphazela kangakanani ubomi bakho?**	0<br>Akukho kuchaphazeleka konke konke	1	2	3	4	5	6	7	8	9	10<br>Buchaphazela ngokuqatha ubomi bam
**Q28**	**Ucinga ukuba isigulo sakho siya kuqhubeka ixesha elingakanani?**	0<br>Ixesha elifutshane kakhulu	1	2	3	4	5	6	7	8	9	10<br>Ngonaphakade
**Q29**	**Uziva unolawulo olungakanani kwisigulo sakho**	0<br>Akukho lulawulo ngokupheleleyo	1	2	3	4	5	6	7	8	9	10<br>Ulawulo olugqithisileyo
**Q30**	**Ucinga ukuba unyango lwakho lungasinceda kangakanani isigulo sakho?**	0<br>Unotshe	1	2	3	4	5	6	7	8	9	10<br>Iluncedo kakhulu
**Q31**	**Uzifumana kangakanani iimpawu zesigulo sakho?**	0<br>Akukho zimpawu kwaphela	1	2	3	4	5	6	7	8	9	10<br>Iimpawu ezininzi ezinzima
**Q32**	**Ukhathazeke kangakanani ngesigulo sakho?**	0<br>Ayinangxaki kwaphela	1	2	3	4	5	6	7	8	9	10<br>Ndikhathazeke kakhulu
**Q33**	**Uvakalelwa kangakanani ukuba uyasiqonda isigulo sakho?**	0<br>Andiqondi konke konke	1	2	3	4	5	6	7	8	9	10<br>Qonda ngokucacileyo
**Q34**	**Isigulo sakho sikuchaphazela kangakanani ngokweemvakalelo? (umz. ingaba ikwenza ube nomsindo, woyike, ukhathazeke okanye udandatheke?)**	0<br>Ayichaphazelekanga kwaphela ngokweemvakalelo	1	2	3	4	5	6	7	8	9	10<br>Uchaphazeleke kakhulu ngokweemvakalelo
**Q35**	**Dwelisa ngokwezikhundla ezona zinto zintathu zibalulekileyo**	Ezona zizathu zibalulekileyo kum zezi:<br>1. ________________________________<br>2. ________________________________<br>3. ________________________________										

	**okholelwa ukuba zibangele <u>isigulo sakho.</u>**	

<u>ICANDELO 4</u>

Eli candelo lineengxelo ezenziwe ngabanye abantu malunga namayeza.
Nceda uphendule le mibuzo ngokusekelwe kwingxaki <u>enye</u> yezempilo ekukhathazayo kakhulu kwi-Q24. Akukho mpendulo zichanekileyo okanye zingalunganga. Ngamava akho abalulekileyo.
Nceda uphawule ibhokisi enye kwinto nganye ehambelana kakuhle nombono wakho:

**Q36**	**Impilo yam okwangoku ixhomekeke kumayeza am**	Ndivuma kakhulu ☐	Vuma ☐	Ukungaqiniseki ☐	Andivumi ☐	Andivumi kakhulu ☐
**Q37**	**Kuyandikhathaza ukusela amayeza**	Ndivuma kakhulu ☐	Vuma ☐	Ukungaqiniseki ☐	Andivumi ☐	Andivumi kakhulu ☐
**Q38**	**Ubomi bam ngebungenakwenzeka ngaphandle kwamayeza am**	Ndivuma kakhulu ☐	Vuma ☐	Ukungaqiniseki ☐	Andivumi ☐	Andivumi kakhulu ☐
**Q39**	**Ngaphandle kwamayeza am ndingagula kakhulu**	Ndivuma kakhulu ☐	Vuma ☐	Ukungaqiniseki ☐	Andivumi ☐	Andivumi kakhulu ☐
**Q40**	**Ngamanye amaxesha ndiye ndikhathazeke ngeziphumo zexesha elide zamayeza am**	Ndivuma kakhulu ☐	Vuma ☐	Ukungaqiniseki ☐	Andivumi ☐	Andivumi kakhulu ☐
**Q41**	**Iyeza lam liyimfihlakalo kum**	Ndivuma kakhulu ☐	Vuma ☐	Ukungaqiniseki ☐	Andivumi ☐	Andivumi kakhulu ☐
**Q42**	**Impilo yam kwixesha elizayo iya kuxhomekeka kumayeza am**	Ndivuma kakhulu ☐	Vuma ☐	Ukungaqiniseki ☐	Andivumi ☐	Andivumi kakhulu ☐
**Q43**	**Iyeza lam liyabuphazamisa ubomi bam**	Ndivuma kakhulu ☐	Vuma ☐	Ukungaqiniseki ☐	Andivumi ☐	Andivumi kakhulu ☐
**Q44**	**Ngamanye amaxesha ndiye ndikhathazeke ngokuxhomekeke kakhulu kumayeza am**	Ndivuma kakhulu ☐	Vuma ☐	Ukungaqiniseki ☐	Andivumi ☐	Andivumi kakhulu ☐
**Q45**	**Amayeza am andikhusela ekubeni ndibe mandundu**	Ndivuma kakhulu ☐	Vuma ☐	Ukungaqiniseki ☐	Andivumi ☐	Andivumi kakhulu ☐

Le mibuzo ilandelayo imalunga neembono zakho malunga namayeza ngokubanzi.

Ezi ziingxelo ezenziwe ngabanye abantu malunga namayeza ngokubanzi.

Nceda uphawule ibhokisi enye ngento nganye ehambelana kakuhle nembono yakho. Akukho mpendulo zichanekileyo okanye zingalunganga.

**Q46**	**Oogqirha basebenzisa amayeza amaninzi**	Ndivuma kakhulu ☐	Vuma ☐	Ukungaqini seki ☐	Andivum i ☐	Andivumi kakhulu ☐
**Q47**	**Abantu abathatha amayeza kufuneka bayeke unyango lwabo okwethutyana rhoqo kwaye kwakhona.**	Ndivuma kakhulu ☐	Vuma ☐	Ukungaqini seki ☐	Andivum i ☐	Andivumi kakhulu ☐
**Q48**	**Amayeza amaninzi ayakhobokisa.**	Ndivuma kakhulu ☐	Vuma ☐	Ukungaqini seki ☐	Andivum i ☐	Andivumi kakhulu ☐
**Q49**	**Amayeza endalo akhuselekile kunamayeza**	Ndivuma kakhulu ☐	Vuma ☐	Ukungaqini seki ☐	Andivum i ☐	Andivumi kakhulu ☐
**Q50**	**Amayeza enza umonakalo omkhulu kunokulunga.**	Ndivuma kakhulu ☐	Vuma ☐	Ukungaqini seki ☐	Andivum i ☐	Andivumi kakhulu ☐
**Q51**	**Onke amayeza ayityhefu**	Ndivuma kakhulu ☐	Vuma ☐	Ukungaqini seki ☐	Andivum i ☐	Andivumi kakhulu ☐
**Q52**	**Oogqirha bathembela kakhulu kumayeza**	Ndivuma kakhulu ☐	Vuma ☐	Ukungaqini seki ☐	Andivum i ☐	Andivumi kakhulu ☐
**Q53**	**Ukuba oogqirha bebenexesha elininzi nezigulane, bebeya kumisela amayeza ambalwa.**	Ndivuma kakhulu ☐	Vuma ☐	Ukungaqini seki ☐	Andivum i ☐	Andivumi kakhulu ☐

<u>ICANDELO LESI -5.</u>

Eli candelo libuza izimvo zakho malunga nokwenza izigqibo zonyango kunye nogqirha wakho ngengxaki yempilo ekukhathazayo kakhulu ukusuka ku-Q24.
Athini amava akho ngokwenza esi sigqibo sonyango nogqirha wakho?
Kwingxelo nganye, phawula kwibhokisi engqinelana kakuhle ukuba uvumelana kangakanani nayo :

		Vuma Ngokungqongqo	Vuma	Andiqiniseka nga	Andivumi	Andivumi Kakhulu
	Ugqirha…					
Q54	ndithethe ngezinye iindlela endizikhethileyo	☐	☐	☐	☐	☐
Q55	ndithethe malunga nokuba kukho eyona ndlela ilungileyo kwezonyango kwingxaki yam yempilo	☐	☐	☐	☐	☐
Q56	banike izimvo zabo malunga nokhetho	☐	☐	☐	☐	☐
Q57	ucele izimvo zam malunga nokhetho	☐	☐	☐	☐	☐
	ndithethile nge...					
Q58	yintoni ebibalulekile kum ngesi sigqibo	☐	☐	☐	☐	☐
Q59	kutheni enye inketho indifanela ngcono kunomnye	☐	☐	☐	☐	☐
Q60	imingcipheko kunye neenzuzo zokhetho kum kunye nempilo yam	☐	☐	☐	☐	☐
	Ndive…					
Q61	kuya kulungile ukukhetha nayiphi na inketho ebesithetha ngayo	☐	☐	☐	☐	☐
Q62	isigqibo esenziwayo yaba sesona silungileyo kum	☐	☐	☐	☐	☐
Q63	noGqirha savumelana ukuba yeyiphi eyona ilungileyo kum	☐	☐	☐	☐	☐

Nceda uphawule le bhokisi ukuba uyakuvuyela ukuba nodliwano-ndlebe olulandelayo olusekelwe kwiimpendulo ozinikileyo apha ngasentla ☐

Enkosi ngokuthatha inxaxheba kolu vavanyo.
Iimpendulo zakho ziya kusetyenziswa liqela lezonyango apha ukuphucula iinkonzo zabo.
