# Supplementary material for: Patient Beliefs and Experiences of Adhering to Medical Therapies for Cardiovascular Comorbidities, Before Noncardiac Elective Surgery in South Africa: A Mixed-Methods Study
Source: Anesth Analg. 2026 Jul 3;143(6):1335–43. doi: 10.1213/ANE.0000000000008110 (PMC13747293; doi:10.1213/ANE.0000000000008110)
Supplement: Supplementary file 2 [file ane-143-1335-s002.pdf]

Full Title: Patient beliefs and experiences of adhering to medical therapies for cardiovascular comorbidities, before non-cardiac elective surgery in South Africa: a mixed-methods study.

Table S4: Summary of themes and subthemes with example quotations

**Theme 1: Understanding illness**	**Example quote**
1.1 Misunderstanding cause of condition	*"I was blown up underground in a goldmine. It blasted and that shock gave me diabetes" 65, M*
1.2 Lifestyle cause	*"Party, wrong eating, go buy your Gatsby, eat them, chips, wrong lifestyle, lack of exercise" 52, M*
1.3 Hereditary cause	*"It's hereditary. My father had it [diabetes]. My uncles have it. Grandfather had it. I think my sister now" – 53, M*
1.4 Permanency of condition	*"I'm going to have diabetes for the rest of my life" –73, M*
1.5 Fear of disease progression	*"…people would lose feet and toes and all sorts…when it was out of control. And I'm very concerned. I don't want any of that to happen" - 73, M*
1.6 Experience of disease progression (symptom perception)	*"I've lost all sensation in my legs…I can't walk far… "-53 M*
**Theme 2: Understanding treatment**	
2.1 Understanding risks without medication	*"If I don't take my medication I know I won't survive" –39, F*
2.2 Not taking medication as directed	*"Sometimes I might not eat sufficiently when I inject…and I get a hypo. That is very dangerous" -73, M*
2.3 Understand how to take medication	*"You can't inject without eating" -73, M*
2.4 Consequences of taking medication	*"If you take it for long, it will affect your kidneys" - 52, M*
2.5 Self-monitoring	*"It's no use you're taking the medicine, and not checking up on you [blood sugar]…I don't have the machine…I must go to the day hospital to order me one there. Because that's a necessity to have- 52, M*
2.5 Patient autonomy for treatment	*"I don't want to rely too much on the medication…I take two tablets then I take one instead of two -[reason] …all the chemical stuff, you know, it's dangerous"- 67, M*

2.6 Understanding role of medications	*“I don’t know [what caused my diabetes…I think it’s that that actually bothers me a lot about it...I’m very uncertain about this disease that I have – 55, F*
2.7 Preference for natural medicines	*“Those natural medicines it gets in your system. It gets much easier because it’s all nature inside…the other medications is chemical, there’s other stuff...it can mess you up” -67, M*
**Theme 3: Role of Health professional**	
3.1 Trusting health professionals	*“It’s basically trial and error…sometimes you think that maybe the pharmaceutical companies are pushing the drug, pushing it onto the doctors” -53, M*
3.2 Time with health professionals	*“There’s no time. It’s all about how many patients can I see a day” – 53, M*<br><br>*“I know there’s a shortage of doctors and they can’t [spend more time with patients] but in an ideal world it would be better. It’s not just the prescribing or overprescribing, it’s to go deeper into the problem.” -65, M*
3.3 Poor explanations from health professionals	*“There’s no depth to the explanations…so unless you read up and go overseas, you’re not going to know” - 65, M*
3.4 Health professional overreliance on medication	*“They bombard you with medicine -52, M*<br>*They must spend more time with patients so that they can…hear the patient’s problems and …[not] just give the medicine, medicine, medicine” – 64, M*
3.5 Medical paternalism	*“Every time you go to the doctor, then he change your medicine. Which is not right, you can’t just change medicine to medicine” - 64, M*
3.6 Patient concerns addressed	*“Every time I go to see a doctor [at the day hospital] then I see a sister or a matron …so I never get to speak to the doctor and tell him how I feel…” -64, M*
**Theme 4: Services and processes**	
4.1 Chronic medication	*“If they put me on chronic medication, then I can go and register at the clinic and get my one dose, and then from there, there are certain pharmacies that you can go direct to it. That will be easier and it cuts all the queues and all the nonsense. They also deliver..” - 65, M*

4.2 Easy access to medication	*"They can bring it to the community center…that's about 5 kilometers…every month I can go fetch it there" - 64, M*
4.3 Home medication service	*"I'm at home. They walk around [and bring it]" -85, F*
4.4 Waiting times	*"At least six to eight hours. The thing is you've got to go early in the morning. You get there at about five o'clock in the morning. You're lucky if you're out by two or three o'clock. It's quite a wait. There are also no facilities. You can't buy any cold drink or food or anything. It's pretty difficult".- 65, M*
4.5 Cost of services	*"He's [private doctor] also become so expensive now, I can't really afford it" - 65, M*
**Theme 5: Accessing hospital**	
5.1 Relying on others	*"My brother normally takes me or a friend of mine…otherwise then I take a taxi…I can count on my brother…" -64, M*
5.2 Access to own transport	*"[distance is] about 15 mins…I drive" – 68, F*
5.3 Cost of transport	*"I must take a taxi...it's half an hour's drive…I must make a plan for the money…I go to my friends and ask them for taxi fare to go to the clinic.." -56, M*
5.4 Safety issues	*"The issue is the transport…it's because of the police, they are standing there. There are a lot of thugs, so it's not easy to go there…I must wait till it's not dark so I can go" -39, F*
**Theme 6: Individual factors**	
6.1 Prioritizing socializing	*"Sometimes you come from a party…and you've still got that buzz in you" – 52, M*
6.2 Intrinsic dislike for medications	*"I am alone, so I have a lot of things on my mind…I just don't like taking medication" -35, F*
6.3 Poor organization	*"If you don't get to the chemist before two o'clock, then you don't have medication for that weekend" - 52, M*
6.4 Expertise	*"I was a nurse, so I know how to take my meds" -68, F*
6.5 Experiencing symptoms	*"…just feels I am dizzy. And then I remember I must take my medication" – 39, F*
6.6 Lifestyle changes	*"I must quit the cigarettes" -56, M*<br>*"Only in the last couple of years have I been really strict about what I put in my mouth. Sugar, particularly" -73, M*
6.7 Additional expenses	*"He gave me diet food…you know how life is, you can't, it's too expensive. You can't afford to" - 64, M*
**Theme 7: Routine/Habit**	
7.1 Location of medicines	*"In my room…when I stand up, I look straight into it…its always there" - 52, M*

7.2 Proactive organization	*“The whole month’s tablets I put in there [pill organizer] then I know I mustn’t skip” - 85, F*
7.3 Daily routine	*“After I’ve eaten …in the mornings and then I take my meds…I don’t forget because it’s like a routine for me” - 67, M*
7.4 Family reminding	*“My wife’s still on my back the whole time…I didn’t see you take them…why are you…late… “-73, M*
7.6 Family support	*“5 o’clock in the morning I wake up. I see my glass of water standing there. My daughter put my water. She knows when mom wakes up, it’s pill time…I take my tablets every morning” – 72, F*
